# Physico-Chemical Characterization, Bioactive Compounds and Antioxidant Activity of Malay Apple [*Syzygium malaccense* (L.) Merr. & L.M. Perry]

**DOI:** 10.1371/journal.pone.0158134

**Published:** 2016-06-28

**Authors:** Polyana Campos Nunes, Jailane de Souza Aquino, Ismael Ivan Rockenbach, Tânia Lúcia Montenegro Stamford

**Affiliations:** 1 Department of Nutrition, Centre for Health Sciences, Federal University of Pernambuco–UFPE, Recife, Pernambuco, Brazil; 2 Department of Nutrition, Centre for Health Sciences, Federal University of Paraíba–UFPB, João Pessoa, Paraíba, Brazil; 3 Department of Food Technology, Centre for Technology and Regional Development, Federal University of Paraíba–UFPB, João Pessoa, Paraíba, Brazil; College of Agricultural Sciences, UNITED STATES

## Abstract

The purpose of this study was to evaluate the physico-chemical characteristics, bioactive compounds and antioxidant activity of Malay apple fruit (*Syzygium malaccense*) grown in Brazil with regard to the geographical origin and its peel fractions and edible portion analyzed independently. Fruit diameter, weight, yield, and centesimal composition, ascorbic acid, reductive sugars, total soluble solids, pH and fiber content were determined. Total phenolics (1293 mg gallic acid equivalent/100 g) and total anthocyanins (1045 mg/100 g) contents were higher in the peel, with the major anthocyanin identified using HPLC-DAD-MS/MS as cyanidin 3-glucoside. Higher values for DPPH antiradical scavenging activity (47.52 μMol trolox equivalent antioxidant capacity/g) and Ferric Reducing Antioxidant Potential (FRAP, 0.19 mM ferreous sulfate/g) were also observed in the peel fraction. All extracts tested showed the ability to inhibit oxidation in the β-carotene/linoleic acid system. This study highlights the potential of Malay apple fruit as a good source of antioxidant compounds with potential benefits to human health.

## Introduction

Many epidemiological studies have been associated with the intake of natural antioxidants present in foods, such as fruits, vegetables and teas with protective effects against many diseases [1,2]. Reddish and purplish coloring of fruits are potential sources of antioxidants, such as phenolics. Among the phenolic, anthocyanins are the main pigments responsible for most of the colors of fruits and exhibit anti-inflammatory, antimutagenic and cancer chemopreventative activities in vivo [3].

A variety of these richly colored fruits is available but has not been investigated for their functional properties though these fruits have been consumed for centuries. Among these fruits, the Malay apple is very popular in Brazil, where this plant can be found in the North, Northeast and Southeastern regions of the country. The fruits are sold in local markets and along the streets wherever the tree is grown. The ripe fruit of Malay apple is eaten raw but can also be eaten with cream for dessert [4].

There has been no report in the international literature regarding the antioxidant activity of Malay apple fruit grown in Brazil. In addition, the physical and chemical characterization of Malay apple fruit is important to support information regarding the nutritional value, proper selection, processing and shelf life of this fruit, which is aimed to encourage the consumption of this fruit because of its potential health benefits as well as to encourage greater application in the food industry. Considering these aspects, this study presents a screening of the antioxidant activity in the peel and edible fraction of the fruit, along with the physical and chemical characterization of *Syzygium malaccense* from different geographical origins.

## Materials and Methods

### Chemicals and samples

The following chemicals were purchased from Sigma-Aldrich Chemie (Steinheim, Germany): 2,2-diphenyl-1-picrylhydrazyl (DPPH), sodium carbonate (Na_2_CO_3_), β-carotene, linoleic acid, BHT (2,6-di-tert-butyl-4-methylphenol), Trolox (6-hydroxy-2,5,7,8-tetramethylchroman-2-carboxylic acid). Cianidin-3-glucoside was purchased from Polyphenols Laboratories AS (Sandnes, Norway). Folin-Ciocalteau reagent, 2,4,6-Tris(2-pyridyl)-1,3,5-triazine (TPTZ) and Tween 40 were purchased from Fluka Chemie AG (Buchs, Switzerland). The solvents employed for extraction and HPLC procedures were analytical/HPLC grade and purchased from Merck (São Paulo, Brazil).

Malay apple samples were obtained from two different geographical origins (designated as L1 and L2), during the harvest season in 2013. Geographic coordinate L1: Latitude 7° 15’ 38” South and Longitude 34° 54’ 30” West. Geographic coordinate L2: Latitude 7° 5’ 39” South and Longitude 59° 54’ 08” West. The field studies did not involve endangered or protected species. The samples collected at locations along public roads for which specific permission is not required were transported to the Biochemistry and Applied Microbiology Laboratory of the Federal University of Paraíba and were then selected and washed in running water for 15 min.

Initially, fruits *in natura* from each L1 and L2 were separated for physical and chemical characterization. Samples were peeled and separated from the seed using a stainless steel knife, yielding two fractions: peel and edible portion (pulp + peel).

For anthocyanins, total phenolic content and antioxidant activity assays; samples were frozen (-80°C) and then lyophilized at -68°C at 130 mmHg in the lyophilizer model Liotop, L101 (São Carlos, Sao Paulo, Brazil) for seven days. After lyophilization, both fractions were separately homogenized in an industrial stainless steel blender with 2 L capacity (Tron), with a velocity of 25.000 rpm when empty and maintained under -30°C in vacuum conditioning until extract preparation.

### Physical and Chemical characterization

The transverse diameter (total height of fruit) and longitudinal diameter were measured using a vernier caliper. Fruit shape (format index) was determined by the ratio of fruit length to diameter. Fruit, pulp, peel and seed weight were measured using a digital balance with an accuracy of 0.01. Peels and seeds were manually separated from the pericarp/membrane, and the total weight per fruit was recorded. Peel yield (%) was determined as the ratio between peel weight and fruit weight, multiplied by 100. Similarly, pulp yield (%) was determined as the ratio between pulp weight and fruit weight, multiplied by 100.

All chemical composition analyses were performed in triplicate. The moisture, ash, fat, protein, fiber and total carbohydrate were quantified. The energy value was calculated from the protein, total lipid and total carbohydrate content using an Atwater system. The total soluble solids (TSS) were determined using an Abbe refractometer (A. Krüss Optronic, Germany) with results expressed as°Brix (at 20°C), and pH was measured using a digital Mettler Toledo MP 230 pH meter (at 20°C) [5].

### Extract preparation

Extracts of the peel and edible fraction from *Syzygium malaccense* were prepared separately. Anthocyanins were exhaustively extracted from 2.0 g of homogenized fractions using methanol containing 1% HCl. Extracts for total phenolic compounds were prepared from 5.0 g of samples, with methanol/water (8:2, v/v), which was also used to evaluate the antioxidant properties. All extracts (anthocyanins and phenolic compounds) were obtained by stirring in a Metabo GE700 homogenizer (Nürtingen, Germany), followed by vacuum filtration. The extracts were concentrated in a rotary evaporator (T < 35°C) and stored in nitrogen at −36°C. Before liquid chromatography analysis (HPLC-DAD-MS/MS), the anthocyanin extract was partially purified on a XAD-7 column (Sigma) to remove sugars.

### Total monomeric anthocyanins

The monomeric anthocyanin contents were determined in both the peel and edible portion extracts using the differential pH method [6]. The monomeric anthocyanin (MA) content was calculated as the equivalent of cyanidin 3-glucoside (cyd 3-glu), considering the molecular weight (MW) of 449.2 g/mol and molar absorption coefficient (ε) of 26,900 L/mol/cm.

### Identification of anthocyanins by HPLC-DAD-MS/MS

Anthocyanins from peel extract were separated on a C18 Shim-pack CLC-ODS column (5 μm, 250 × 4.6 mm i.d.) (Shimadzu, Canby, USA) using a linear gradient of water/methanol as the mobile phase, both with 5%_v/v_ formic acid from 90:10 to 60:40 in 20 min, and passed to 20:80 in 15 min while maintaining this proportion for 5 min, according to a previously described method by Faria et al. [7]. The UV–Vis spectra were acquired between 200 and 600 nm, and the chromatograms were processed at 520 nm. After passing through the cell of the DAD, the flow from the column was split, allowing only 0.15 mL/min into the ESI source. For MS detection, the ESI ionization source was used in either positive (ESI^+^) or negative (ESI^−^) modes, with the capillary voltage at 2.5 and 1.5 kV, respectively. The output voltage of the capillary was 95.2 V for anthocyanins (ESI^+^). The other conditions were as follows: end plate offset −500 V, drying gas (N_2_) temperature of 325°C and flow of 8 L/min, nebulizer at 30 psi. The MS/MS was acquired in automatic mode, applying fragmentation energy of 1.2 V. The scan range was from m/z 100 to 1000.

### Total phenolic compounds and antioxidant activity

#### Total phenolic compounds content

The determination of total phenolic compounds was performed by the reaction with Folin-Ciocalteau reagent [8]. Quantification was performed based on the calibration curve of gallic acid (2.0–8.0 mg/L), and the results were expressed in mg gallic acid equivalent (GAE)/100 g sample.

#### Antioxidant activity–DPPH Method

The DPPH method was performed as described by Brand-Williams et al. [9]. The decrease in the absorbance of 100 μM DPPH• radicals (2.9 mL) dissolved in 80% methanol was evaluated at 515 nm, 30 min after the addition of each extract. The total antioxidant activity of peel and edible portion (on a dry weight basis) were expressed in μMol/g of TEAC.

#### Reducing Power–FRAP Method

The ferric reducing antioxidant potential (FRAP) assay was performed according to the method described by Benzie and Strain [10], based on the direct measurement of antioxidant (reducing) ability through the reduction of the complex Fe^3+^/tripyridyltriazine (TPTZ) to Fe^2+^ at acid pH (3.6). The absorbance was read at 620 nm using a UV–vis spectrophotometer during the monitoring period (2 h). The antioxidant potential of the peel and edible portion extracts was determined based on a calibration curve plotted using FeSO_4_∙7H_2_O at a concentration ranging between 500 and 2000 μM.

### Oxidation inhibition power–Co-oxidation of β-carotene/linoleic acid method

The oxidation inhibition power of the fruit methanolic extract was evaluated as described by Marco [11] and Rodrigues et al. [12], with some modifications: a 20-μL aliquot of β-carotene solution (20 mg/mL in chloroform) was placed in a 250 mL Erlenmeyer flask with 40 μL of linoleic acid, 1 mL chloroform and 20 mg of Tween 40. After homogenization, the chloroform was completely evaporated with nitrogen. Deionized water (previously subjected to oxygen atmosphere for 30 min) was then added until the formation of a clear emulsion with absorbance ranging from 0.6 to 0.7 at 470 nm. Different aliquots of the extracts (100 μL and 400 μL), BHT (Butylated hydroxytoluene, 100 μL) and a mixture of extracts (50 μL) + BHT (50 μL) were added to 5 mL of linoleic acid emulsion in glass cuvettes (optical path of 10 mm). After the initial reading, the absorbance was monitored every 15 min for 2 h. During this period, the cuvettes were incubated at 50°C in a water bath. The decrease in absorbance was compared to the control (without antioxidant). The synergistic effect was also evaluated using 50 μL of the extracts and BHT at a 1:1 ratio. The antioxidant activity was expressed as % inhibition, relative to the control, according to Eq 1.

$$\text{inhibition}\;\%=100-\left(\frac{A_{i}-A_{f}}{C_{i}-C_{f}}x\;100\right)$$ (1)

Ai = extract initial absorbance

Af = extract final absorbance

Ci = control initial absorbance

Cf = control final absorbance

### Statistical Analysis

Data were subjected to ANOVA and Student’s t-test (*Statistica*® 7.0 software) at a 0.01 level of significance. All assays being performed in triplicate.

## Results

### Physico-chemical characterization

The physical characteristics of *Syzygium malaccense* are summarized in Table 1. Fruits from L2 were slightly larger and heavier than those from L1 (p<0.05). The weight of the fruits, on average, was 62.21 g and measured 5.91 cm longitudinally in diameter and 4.87 cm transversely in diameter.

**Table 1:** Physical characteristics^*^ of *Syzygium malaccense* obtained from different geographical origins (L1 and L2). Mean ± standard deviation followed by different letters in the same row differ according to Student’s t-test (*p<*0.01).

Characteristics	L1	L2
Transverse diameter (TD) (cm)	4.81 ± 0.32 ^b^	4.92 ± 0,4 ^a^
Longitudinal diameter (LD) (cm)	5.89 ± 0.39 ^b^	5.93 ± 0,39 ^a^
Format index LD/TD	1.24 ± 0.09 ^a^	1.20 ± 0,09 ^b^
Fruit weight (g)	60.86 ± 7.27 ^b^	63.56 ± 8.78 ^a^
Pulp weight (g)	38.80 ± 4.31 ^b^	40.93 ± 7.22 ^a^
Peel weight (g)	9.97 ± 2.62 ^b^	11.52 ± 3.68 ^a^
Seed weight (g)	12.09 ± 3.53 ^a^	11.11 ± 3.61 ^b^
Peel yield (%)	16.34 ± 3.30 ^b^	18.19 ± 5.47 ^a^
Pulp yield (%)	64.02 ± 4.75 ^b^	64.32 ± 5.81^a^

*Mean for 50 fruits

There were significant differences (*p*<0.01) in the physical and chemical characteristics between the fractions (peel and edible portion) of samples from the same geographical origin and between the same fractions considering its different geographical origin (Table 2). On the basis of these results, the peels presented higher contents of carbohydrates, lipids, ashes, ascorbic acid, reductive sugars and total soluble solids, and lower values of moisture, proteins, pH and food fiber when compared to the edible portion.

**Table 2:** Physico-chemical characterization of *Syzygium malaccense* obtained from different geographical origins (L1 and L2). Mean ± standard deviation of triplicate measurement followed by different letters in the same row differ according to Student’s t-test (*p<*0.01). Small letters compare different fractions (peel and edible fruit) of the same geographical origin. Capital letters compare the same fractions (peel or edible fruit) of different geographical origin (L1 and L2).

Composition (per 100 g)	L1		L2	
	Peel	Edible portion	Peel	Edible portion
Moisture (g)	89.28 ± 0.21 ^bB^	91.42 ± 0.73 ^aA^	90.03 ± 0.24 ^bA^	90.93 ± 0.45 ^aB^
Ash (g)	0.52 ± 0.23 ^aA^	0.37 ± 0.17 ^bA^	0.41 ± 0.22 ^aB^	0.25 ± 0.06 ^bB^
Protein (g)	0.82 ± 0.46 ^bA^	1.04 ± 0.36 ^aA^	0.59 ± 0.29 ^bB^	0.72 ± 0.22 ^aB^
Lipids (g)	0.30 ± 0.05 ^aA^	0.19 ± 0.11 ^bA^	0.24 ± 0.06 ^aB^	0.12 ± 0.02 ^bB^
Fiber (g)	1.30 ± 0.78 ^bB^	1.93 ± 0.49 ^aA^	1.42 ± 0.55 ^bA^	1.50 ± 0.66 ^aB^
Carbohydrates (g)	7.78 ± 1.56 ^aA^	5.05 ± 0.73 ^bB^	7.31 ± 0.81 ^aB^	6.48 ± 1.18 ^bA^
Energy (kcal)	37.10 ± 2.93 ^bB^	26.07 ± 2.52 ^aB^	33.76 ± 1.30 ^bA^	29.88 ± 1.59 ^aA^
Ascorbic acid (mg)	318.01 ± 0.77 ^aB^	171.14 ± 1.61 ^bB^	321.27 ± 1.18 ^aA^	175.06 ± 1.09 ^bA^
Reductive Sugars (g)	0.66 ± 0.04 ^aA^	0.52 ± 0.36 ^bB^	0.61 ± 0.36 ^aB^	0.56 ± 0.20 ^bA^
Total Soluble Solids (°Brix)	6.76 ± 0.00 ^aA^	5.46 ± 0.44 ^bB^	6.5 ± 0.36 ^aB^	6.02 ± 0.47 ^bA^
pH	3.60 ± 0.01 ^bA^	3.75 ± 0.02 ^aB^	3.54 ± 0.01 ^bB^	3.82 ± 0.02 ^aA^

### Anthocyanins

**Fig 1:**
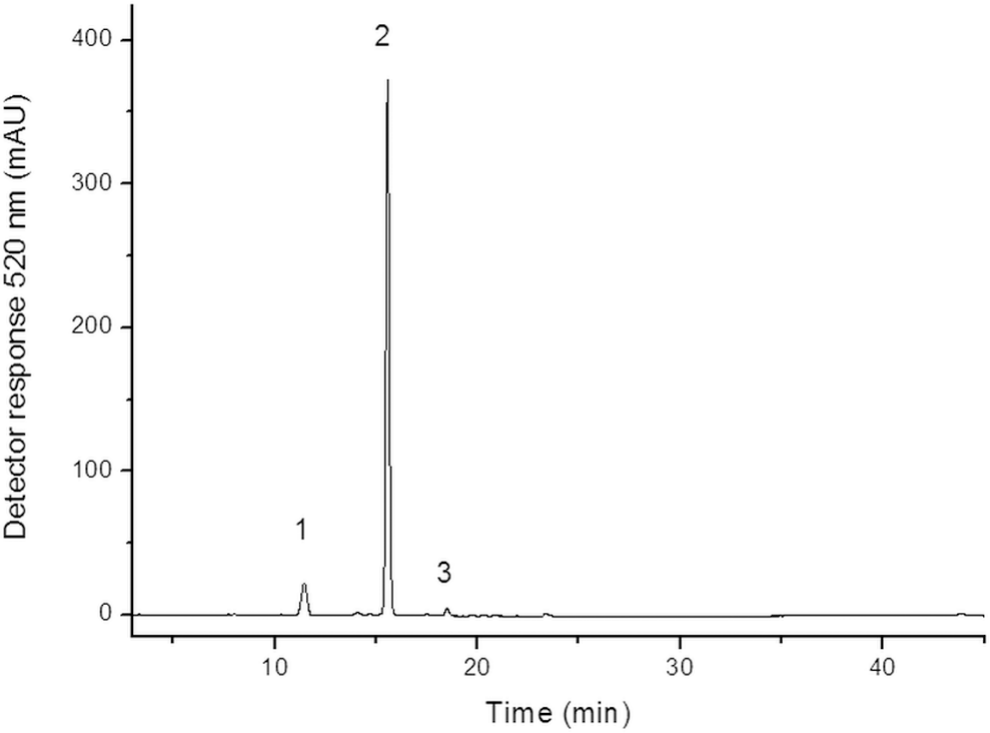
Chromatogram, obtained by HPLC–DAD–MS/MS, of anthocyanins from *Syzygium malaccense*. Peak 1 = cyanidin 3,5-diglucoside; Peak 2 = cyanidin 3-glucoside; Peak 3 = peonidin 3-glucoside.

The chromatogram of the extract of anthocyanins from Malay apple is presented in Fig 1, as measured using HPLC–DAD–MS/MS. The major anthocyanin found in Malay apple was cyanidin 3-glucoside, followed by cyanidin 3,5-diglucoside and peonidin 3-glucoside (Table 3). These results are consistent with those reported in previous studies with Malay apple fruits, where the major anthocyanins were identified as cyanidin 3-glucoside [3], and these compounds have been shown to protect cell membrane lipids from oxidation [13].

**Table 3:** Anthocyanins identified in *Syzygium malaccence* fruit.

Peak^a^	Compounds	t_R_ (min)^b^	λ_max_ (nm)	[M]^+^ (*m/z*)	MS/MS (*m/z*)
1	cyanidin 3,5-diglucoside	11.44	517	611	449[M-162]^+^, 287 [M-162-162]^+^
2	cyanidin 3-glucoside	15.4	514	449	287 [M-162]^+^
3	peonidin 3-glucoside	18.5	517	463	301 [M-162]^+^

^a^ numbered according to Fig 1.

^b^ retention time (min).

### Total phenolic compounds and antioxidant activity

Total phenolic compounds content and antioxidant activity were lower in the edible portion of fruit when compared to the peel fraction (Table 4). There were significant differences (*p*<0.01) between all of the parameters compared, except for the FRAP assay, when comparing the same fraction (peel and edible portion) in samples obtained from different geographical origin (L1 and L2).

**Table 4:** Total Phenolic Compounds (TPC) and Antioxidant Activity (DPPH and FRAP) of extracts of peel and edible portion from *S*. *malaccense* obtained from different geographical origins (L1 and L2). Mean ± standard deviation of triplicate measurements followed by different letters in the same row differ according to Student’s t-test (*p*<0.01). Small letters compare different fractions (peel and edible fruit) of the same geographical origin. Capital letters compare the same fractions (peel or edible fruit) of different geographical origin (L1 and L2).

Samples	L1		L2	
	Peel	Edible portion	Peel	Edible portion
TPC^1^	12.58 ± 0.03 ^aB^	8.03 ± 0.67 ^bB^	12.93 ± 0.02 ^aA^	7.64 ± 0.02 ^bA^
DPPH^2^	47.22 ± 0.26 ^aB^	25.92 ± 0.28 ^bA^	47.52 ± 0.41 ^aA^	22.20 ± 0.50 ^bB^
FRAP^3^	0.19 ± 0.01 ^aA^	0.09 ± 0.00 ^bA^	0.19 ± 0.02 ^aA^	0.09 ± 0.01 ^bA^

^1^ Expressed as mg GAE (gallic acid equivalent)/g dry weight.

^2^ Expressed as μMol TEAC (Trolox equivalent antioxidant capacity)/g dry weight.

^3^ Expressed as mM ferreous sulfate/g dry weight.

### Oxidation inhibition power (OIP)

**Fig 2:**
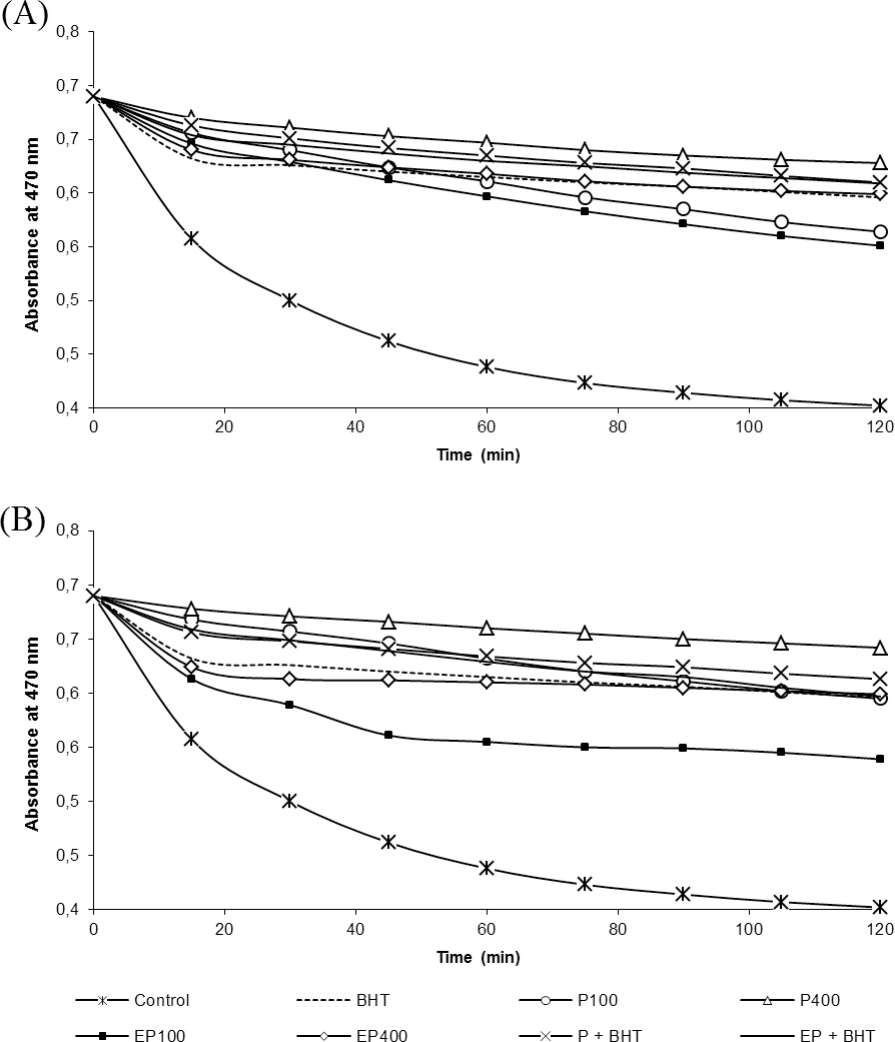
**Oxidation inhibition power of *Syzygium malaccense* from L1 (A) and L2 (B) extracts and BHT.** P100 = 100 μL of peel extract; P400 = 400 μL of peel extract; EP100 = 100 μL of edible portion extract; EP400 = 400 μL of edible portion extract; P + BHT = 50 μL of peel extract + 50 μL of BHT; EP + BHT = 50 μL of edible portion + 50 μL of BHT.

Fig 2 shows the comparative β-carotene oxidation inhibition power of the control, BHT, mixture of extracts + BHT and different aliquots of peel and edible portion extracts from L1 and L2. The decrease of β-carotene absorbance indicates that all tested extracts presented antioxidant capacity. The OIP was in the following order: P400 (78.47%) > P+BHT (72.22%) > EP+BHT (71.88%) > EP400 (68.40%) > BHT (67.36%) > P100 (56.25%) > EP100 (51.74%) for extracts from L1; and P400 (83.33%) > P+BHT (73.26%) > EP400 (68.40%) > EP+BHT (67.71%) > BHT (67.36%) > P100 (67.01%) > EP100 (47.57%) for extracts from L2.

## Discussion

Fruits with a similar weight and uniform shape are desirable in terms of marketing value. In addition, the demand of fruit quality, with regard to its physical and chemical characteristics, by industries is dependent on fruit species and physical characteristics. Among the physical characteristics, dimensions, mass and yield are important parameters in sizing and fruit appearance [14].

The Malay apple is presumed to be a native fruit from Malaysia and was introduced in Brazil and other tropical countries. The morphological and climatic adaptation of Malay apple in different geographical regions may reflect on the physical and chemical characteristics, as it was found in this study. There were significant differences on these aspects between fruits from L1 and L2 regions. These findings are also expected because the physico-chemical characteristics in fruits are dependent on genetic and environmental factors [15] and production practices [16]. According to Costa et al. [17], the epicarp of Malay apple is thin, smooth, and reddish; the mesocarp and endocarp are whitish and succulent. These authors reported that the fruits weighed 35.57 g and measured 7.16 cm in length and 5.15 cm in width for samples originated from Southeast Brazil, showing a great difference when compared to fruits from L1 and L2 regions evaluated in our study.

There is scarce information available in the literature regarding the proximate composition and chemical characteristics of Malay apple fruits (*S*. *malaccense*). Therefore, we will use reference data of other fruits with known composition, which are recognized for their health benefits. The moisture level of both fractions of the fruit was approximately 90%, which lies within the class of fleshy and succulent fruits, a common characteristic in tropical fruits. A similar result of 87% of moisture was reported by Oliveira et al. [18], while 83.28% of moisture was reported by Lim and Rabeta [19], in Malay apple. The carbohydrate level, which is approximately 6.5%, is the highest calorie contributor of the Malay apple and many tropical fruits, particularly in Malay apple, as the total lipid and protein contents do not substantially affect the total energy value of the fruit [19, 20].

It is well documented that vitamin C, a water-soluble vitamin, is a strong antioxidant [21]. A considerable amount of ascorbic acid was found in *S*. *malaccense* peel (318.01 and 321.27 mg/100 g for L1 and L2, respectively). The outcome of this study was consistent with reports obtained from Judprasong et al. [21], which showed high ascorbic acid values in other regional fruits. Satpathy et al. [22] reported 200.2 mg/100 g (dry material) for *Spondias pinnata* K., and Clerici and Carvalho–Silva [20] reported 575 mg/100 g (dry material) for *Phyllanthus emblica* L. In addition, the *S*. *malaccense* peel can be considered a source of vitamin C, to cater to more than 15% of the Dietary Reference Intake–DRI for adults and children [23].

The peel and edible portion samples showed the lowest fiber level compared with other dark-colored fruits, such as blackberries (5.30%), blueberries (2.40%), strawberries (2.00%) and grapes (fresh weight) [24], and cannot be considered fiber sources [25]. In the present study, the peel showed the highest Total Soluble Solids (TSS, in°BRIX) at a range between 6.5 (L2) and 6.76 (L1) and the lowest pH at a range between 3.54 and 3.60 when compared to the edible portion (p<0.05). TSS and pH are important characteristics related with sugar content and quality factor as it balances sweetness and tartness [16]. Furthermore, pH ranges could be affected by degradation as well as by different chemical forms in which anthocyanin are found in foods. At a pH value between 1.0 and 4.0, the flavylium cation (red color) and quinoidal blue species are predominant, and colorations are visually observed in the peel of Malay apple fruit [26].

Although Malay apple fruit peels and pulps are eaten together, the peels present a higher concentration of bioactive compounds, thus, they were analyzed separately in this study. Among bioactive compounds, anthocyanins have been reported to be powerful antioxidant compounds, and stability studies showed that they can be stable for up to 6 months in dry pulps [27]. When compared to other fruits from the Myrtaceae family, Malay apple showed a higher content of monomeric anthocyanins (1032.01 mg/100 g and 278.34 mg/100 g for peel and edible portion, respectively, to extracts from L1 and 1045.34 mg/100 g and 275.68 mg/100 g to extracts from L2) compared to those reported for camu–camu (*Myrciaria dubia*), 30–54 mg/100 g [28], *Eugenia myrtifolia*, 33 mg/100 g [29], and jambolão (*Syzygium cumini*), 134 mg/100 g (fresh weight) [30]. Differences in anthocyanins content can be attributed to the inherent variability of the raw material, as well as to differences in the methodology or standard used.

Further, we have found that cyanidin 3-glucoside is the major anthocyanin in Malay apple fruit. Different studies suggest its beneficial effects to human health. For example, the potential efficacy of this anthocyanin pigment against oxidative damages and the protective effects on oxidative stress, inflammation suppression, obesity prevention, attenuation of lung injury and primary hepatocytes protection [31, 32, 33, 34, 35]. Therefore, the use of dietary sources of cyanidin 3-glucoside through fruits and vegetables consumption may play an important role as a dietary antioxidant and healthy strategy.

Total phenolic compounds content and antioxidant activity of Malay apple can be mainly attributed to the peel’s deep purple color, indicating a rich presence of anthocyanins and other bioactive compounds [36]. In the study of antiradical phenolic constituents of fourteen edible fruits from Myrtaceae family, Reynertson et al. [3] reported a similar result for TPC in *Syzygium malaccense* (8.58 mg GAE/g dry weight for edible portion), which is lower when compared to those for other fruits from the Myrtaceae family (*Myrciaria dubia*, 101.0, *Myrciaria vexator*, 44.1, *Syzygium curranii*, 39.6, *Myrciaria cauliflora*, 31.6, and *Eugenia aggregata*, 25.3 mg GAE/g dry weight). In addition, some authors have quantified the TPC in extracts from leaves of Malay apple [37, 38] with mean results of 8.87 mg GAE/g dry weight [37]. These findings are similar to the value for the edible portion, but the quantification of TPC in the peel is scarce.

Because the redox properties of polyphenols allow them to act as reducing agents, hydrogen donators and singlet oxygen quenchers, as well as metal chelating agents [38], different assays were employed to determine and compare the antioxidant properties of *S*. *malaccense* in the peel and edible portion. The DPPH antiradical scavenging activity values were 47.22 and 47.52 μMol TEAC/g for peel extracts and 25.92 and 22.20 μMol TEAC/g for edible portion extracts in L1 and L2, respectively. These results are similar to the findings obtained by Azevêdo [38] (29.03 μMol TEAC/g) for the edible portion.

The FRAP values were 0.19 (peel) and 0.09 (edible portion) mM ferreous sulfate/g dry weight and were consistent with findings obtained by Ikram et al. [39], who found FRAP values of 0.22 mM for *Syzgiujm malaccense* lyophilized fruits. Lim and Rabeta [19] reported a value of 2.06 mM of ferrous equivalent Fe (II) per gram of freeze-dried sample extracted with 50% acetone. As described by these authors, samples extracted with acetone have a higher FRAP value compared with samples extracted with water [19].

Earlier studies have shown that there is a correlation between antioxidant activity and total phenolic compounds (TPC) in fruits [40, 41]. In the present study, positive correlations (*p*<0.01) between the total phenolic content and DPPH antiradical scavenging activity values (*r* = 0.9424) and between the total phenolic content and FRAP values (*r* = 0.9667) were observed.

In addition to exhibiting good antioxidant activity and significant reducing power, the peel and edible portion extracts of Malay apple also showed a moderate capacity to inhibit the oxidation of the β-carotene/linoleic acid system. Although it was observed that fruits from different geographical origins exhibited a small variation in OIP, all antioxidant extracts tested showed the ability to inhibit oxidation in the β-carotene/linoleic acid system. The same ability to inhibit oxidation was observed in the studies of Ikram et al. [39] and Rockenbach et al. [41], who evaluated extracts of Malaysian under-utilized fruits and pomace from red grapes, respectively. For both L1 and L2, a higher OIP was observed to P400 and P+BHT, demonstrating a higher potential of peel extracts compared to edible portion extracts and a positive synergistic effect of peel extracts and BHT. A hypothesis for the synergistic effect between peel extracts and BHT is based on the higher level of anthocyanins in the peel compared to the edible portion, indicating a high capacity of these compounds to scavenge free radicals liberated during linoleic acid oxidation [40]. Finally, it is also noteworthy that the higher proportion of peel in relationship to the edible portion observed in fruit from geographical origin L2 might be the reason for its higher concentration of anthocyanins, higher antioxidant activity and oxidation inhibition power compared to fruit from L1.

## Conclusions

Fruits from different geographical origins of Brazil presented significant differences (*p*<0.01) in chemical and physical parameters. The peel is a source of vitamin C and contains a higher content of bioactive compounds and antioxidant activity. In addition, peel extracts demonstrated a higher oxidation inhibition power compared to edible portion extracts and a positive synergistic effect between peel extracts and BHT was observed. With regard to the few reports on *Syzygium malaccense*, this study provides new information about the physical and chemical properties, bioactive compounds and antioxidant activity of this fruit.

## References

[1] Xu Y-J, Tappia PS, Neki NS, Dhalla NS. Prevention of diabetes-induced cardiovascular complications upon treatment with antioxidants. Heart Fail Rev. 2014; 19:113–121. 10.1007/s10741-013-9379-6 23436032

[2] Corley J, Kyle JAM, Starr JM, McNeill G, Deary IJ. Dietary factors and biomarkers of systemic inflammation in older people: the Lothian Birth Cohort 1936. Br J Nutr. 2015; 114:1088–109. 10.1017/S000711451500210X 26343358 PMC4579562

[3] Reynertson KA, Yang H, Jiang B, Basile MJ, Kennelly EJ. Quantitative analysis of antiradical phenolic constituents from fourteen edible Myrtaceae fruits. Food Chem. 2008; 109: 883–90. 21340048 10.1016/j.foodchem.2008.01.021 PMC3040238

[4] Morton JF. Malay Apple In: Fruits of warm climates. Miami: Florida Flair Books, 1987, p.378–81.

[5] AOAC. Officials Methods of Analysis 6th ed. Arlington: Association of Official Analytical Chemist, 2002.

[6] Lee J, Durst RW, Wrolstad RE. Determination of total monomeric anthocyanin pigment content of fruit juices, beverages, natural colorants, and wines by the pH differential method: Collaborative Study. J AOAC. 2005; 88: 1269–78. 16385975

[7] Faria AF, Marques MC, Mercadante AZ. Identification of bioactive compounds from jambolão (*Syzygium cumini*) and antioxidant capacity evaluation in different pH conditions. Food Chem. 2011; 126: 1571–8. 10.1016/j.foodchem.2010.12.007 25213929

[8] Singleton VL, Rossi JA. Colorimetry of total phenolics with phosphomolybdic phosphotungstic acid reagents. Am J Enol Vitic. 1965; 16: 144–58.

[9] Brand-Williams W, Cuvelier ME, Berset C. Use of a free radical method to evaluate antioxidant activity. *LWT–*Food Sci Technol. 1995; 22: 25–30.

[10] Benzie IF, Strain JJ. The ferric reducing ability of plasma (FRAP) as a measure of "antioxidant power": the FRAP assay. Anal Biochem. 1996; 239(1):70–6. 8660627 10.1006/abio.1996.0292

[11] Marco GJ. A rapid method for evaluation of antioxidants. J Am Oil Chem Soc.1968; 45: 594–98.

[12] Rodrigues E, Poerner N, Rockenbach II, Gonzaga LV, Mendes CR, Fett R. Phenolic compounds and antioxidant activity of blueberry cultivars grown in Brazil. Cienc Tecnol Aliment. 2011; 31(4): 911–7.

[13] Tsuda T. Diatery cyanidin 3-O-beta-D-glycoside increases in vivo oxidative, resistance of serum in rats. Lipids.1998; 6: 583–8. 10.1007/s11745-998-0243-5 9655373

[14] Khoshnam F, Tabatabaeefar A, Ghasemi Varnamkhasti M, Borghei A. Mass modeling of pomegranate (*Punica granatum* L.) fruit with some physical characteristics. Sci Hort. 2007; 114: 21–6.

[15] Doshi P, Adsule P, Banerjee K. Phenolic composition and antioxidant activity in grapevine parts and berries (*Vitis vinifera* L.) cv. Kishmish Chornyi (Sharad Seedless) during maturation. Int J Food Sci Technol. 2006; 41: 1–9.

[16] Radunić M, Špika MJ, Ban SG, Gadže J, Díaz-Pérez JC, MacLean D. Physical and chemical properties of pomegranate fruit accessions from Croatia. Food Chem. 2015; 177: 53–60. 10.1016/j.foodchem.2014.12.102 25660857

[17] Costa RS, Oliveira IVM, Môro FV, Martins ABG. Morphological aspects and influence of the seed size in the germination of wax jambu. Rev Bras Frutic. 2006; 28: 117–20.

[18] Oliveira FIP, Gallão MI, Rodrigues S, Fernandes FAN. Dehydration of Malay Apple (*Syzygium malaccense* L.) using ultrasound as pre-treatment. Food Bioprocess Technol. 2011; 4: 610–5.

[19] Lim AS, Rabeta MS. Proximate analysis, mineral content and antioxidant capacity of Milk apple, Malay apple and Water apple. 2013; 20(2): 673–9.

[20] Clerici MTPS Carvalho–Silva LB. Nutritional bioactive compounds and technological aspects of minor fruits grown in Brazil. Food Res Int. 2011; 44:1658–70.

[21] Judprasong K, Charoenkiatkul S, Thiyajai P, Sukprasansap M. Nutrients and bioactive compounds of Thai indigenous fruits. Food Chem. 2013; 140: 507–12. 10.1016/j.foodchem.2013.01.057 23601399

[22] Satpathy G, Tuagi YK, Gupta RK. Preliminary evaluation of nutraceutical and therapeutic potential of raw *Spondias pinnata* K., an exotic fruit of India. Food Res Int. 2011; 44: 2076–87.

[23] IOM. Institute of Medicine. Dietary Reference Intakes for vitamin C, vitamin E, selenium and carotenoids Washington DC: National Academy Press, 2001.

[24] USDA. U.S. Department of Agriculture, Agricultural Research Service. USDA National Nutrient Database for Standard Reference, Release 21. Food Group: 09 Fruits and fruit Juices. Available: http://www.ars.usda.gov/ba/bhnrc/ndl. Accessed: 20, November, 2014.

[25] Institute of Medicine. Dietary Reference Intakes for Energy, Carbohydrate, Fiber, Fat, Fatty Acids, Cholesterol, Protein and Amino Acids Washington DC: National Academy Press, 2001. 10.1016/s0002-8223(02)90346-9 12449285

[26] Loypimai P, Moongngarm A, Chottanom P. Thermal and pH degradation kinetics of anthocyanins food colorant prepared from black rice bran in natural. J Food Sci Technol. 2016; 53:461–70. 10.1007/s13197-015-2002-1 26787965 PMC4711436

[27] Banerjee A, Dasgupta N, De B. In vitro study of antioxidant activity of *Syzygium cumini* fruit. Food Chem. 2005; 90: 727–33.

[28] Zanatta CF, Mercadante AZ. Carotenoid composition from the Brazilian tropical fruit camu–camu (*Myrciaria dubia*). Food Chem. 2007; 101: 1526–32.

[29] Longo L, Scardino A, Vasapollo G, Blando F. Anthocyanins from *Eugenia myrtifolia* Sims. Innov Food Sci Emer Technol. 2007; 8: 329–32.

[30] Benherlal PS, Arumughan C. Chemical composition and in vitro antioxidant studies on *Syzygium cumini* fruit. J Sci Food Agric. 2007; 87: 2560–9. 10.1002/jsfa.2957 20836162

[31] Pratheeshkumara P, Sona Y, Wanga X, Divyaa SP, Josephc B, Hitrona JA, Wanga L, Kimb D, Yinb Y, Roya RV, Lub J, Zhangb Z, Wangf Y, Shia X. Cyanidin-3-glucoside inhibits UVB-induced oxidative damage and inflammation by regulating MAP kinase and NF-κB signaling pathways in SKH-1 hairless mice skin. Toxicol Appl Pharm. 2014; 280: 127–37. 10.1016/j.taap.2014.06.028 PMC4330564 25062774

[32] Fu Y, Zhou E, Wei Z, Wang W, Wang T, Yang Z, Zhang N. Cyanidin-3-O-β-glucoside ameliorates lipopolysaccharide-induced acute lung injury by reducing TLR4 recruitment into lipid rafts. Biochem Pharmacol. 2014; 90: 126–34. 10.1016/j.bcp.2014.05.004 24841888

[33] Yan X, Wu L, Li B, Meng X, Dai, H, Zheng Y, Fu J. Cyanidin-3-O-glucoside attenuates acute lung injury in sepsis rats. J Surg Res. 2015; 199: 592–600. 10.1016/j.jss.2015.06.013 26152793

[34] Song J, Zhao M, Liu X, Zhu Y, Hu X, Chen F. Protection of cyanidin-3-glucoside against oxidative stress induced by acrylamide in human MDA-MB-231 cells. Food Chem Toxicol. 2013; 58: 306–10. 10.1016/j.fct.2013.05.003 23685245

[35] Tang L, Li S, Bi H, Gao X. Interaction of cyanidin-3-O-glucoside with three proteins. Food Chem. 2016; 196: 550–59. 10.1016/j.foodchem.2015.09.089 26593527

[36] Arumugam B, Manaharan T, Heng CK, Kuppusamy UR, Palanisamy UD. Antioxidant and antiglycemic potentials of a standardized extract of *Syzygium malaccense*. Food Sci Technol. 2014; 59: 707–12.

[37] Savitha RC, Padmavathy S, Sundhararajan A. *In vitro* antioxidant activities on leaf extracts of *Syzygium Malaccense* (L.) Merr and Perry. Anc Sci Life. 2011; 30: 110–3. 22557439 PMC3336265

[38] Azevêdo JCS. Estratégias de obtenção do corante do jambo vermelho (*Syzygium malaccense*) e avaliação de sua funcionalidade. M.Sc. Thesis, Federal University of Rio Grande do Norte. 2010. Available: http://repositorio.ufrn.br/handle/123456789/15804

[39] Ikram EHK, Eng KH, Jalil AMM, Ismail A, Idris S, Azlan A, et al Antioxidant capacity and total phenolic content of Malaysian underutilized fruits. J Food Compost Anal. 2008; 22: 388–93.

[40] Balasundram N, Sundram K, Samman S. Phenolic compounds in plants and agri-industrial by-products: Antioxidant activity, occurrence, and potential uses. Food Chem. 2006; 99: 191–203.

[41] Rockenbach II, Rodrigues E, Gonzaga LV, Caliari V, Genovese MI, Gonçalves AE de SS, Fett R. Phenolic compounds content and antioxidant activity in pomace from selected red grapes (*Vitis vinifera* L. and *Vitis labrusca* L.) widely produced in Brazil. Food Chem. 2011; 127: 174–9.

